# EgGLUT1 Is Crucial for the Viability of *Echinococcus granulosus sensu stricto* Metacestode: A New Therapeutic Target?

**DOI:** 10.3389/fcimb.2021.747739

**Published:** 2021-11-11

**Authors:** Kuerbannisha Amahong, Mingzhi Yan, Jintian Li, Ning Yang, Hui Liu, Xiaojuan Bi, Dominique A. Vuitton, Renyong Lin, Guodong Lü

**Affiliations:** ^1^ State Key Laboratory of Pathogenesis, Prevention, and Treatment of Central Asian High Incidence Diseases, Clinical Medical Research Institute, The First Affiliated Hospital of Xinjiang Medical University, Urumqi, China; ^2^ College of Pharmacy, Xinjiang Medical University, Urumqi, China; ^3^ French National Reference Centre for Echinococcosis, University Bourgogne Franche-Comté, Besançon, France; ^4^ WHO Collaborating Centre for Prevention and Care Management of Echinococcosis, The First Affiliated Hospital of Xinjiang Medical University, Urumqi, China; ^5^ Basic Medical College, Xinjiang Medical University, Urumqi, China

**Keywords:** *Echinococcus granulosus sensu stricto*, glucose transporter 1, WZB117, cystic echinococcosis, glucose uptake

## Abstract

Cystic echinococcosis (CE) is a zoonotic parasitic disease caused by infection with the larvae of *Echinococcus granulosus sensu lato* (*s.l.*) cluster. It is urgent to identify novel drug targets and develop new drug candidates against CE. Glucose transporter 1 (GLUT1) is mainly responsible for the transmembrane transport of glucose to maintain its constant cellular availability and is a recent research hotspot as a drug target in various diseases. However, the role of GLUT1 in *E. granulosus s.l.* (EgGLUT1) was unknown. In this study, we cloned a conserved GLUT1 homology gene (named EgGLUT1-ss) from *E. granulosus sensu stricto* (*s.s.*) and found EgGLUT1-ss was crucial for glucose uptake and viability by the protoscoleces of *E. granulosus s.s.* WZB117, a GLUT1 inhibitor, inhibited glucose uptake by *E. granulosus s.s.* and the viability of the metacestode *in vitro*. In addition, WZB117 showed significant therapeutic activity in *E. granulosus s.s.*-infected mice: a 10 mg/kg dose of WZB117 significantly reduced the number and weight of parasite cysts (*P* < 0.05) as efficiently as the reference drug, albendazole. Our results demonstrate that EgGLUT1-ss is crucial for glucose uptake by the protoscoleces of *E. granulosus s.s.*, and its inhibitor WZB117 has a therapeutic effect on CE.

## Introduction

Cystic echinococcosis (CE) is a chronic and neglected zoonotic parasitic disease caused by the larvae of the *Echinococcus granulosus sensu lato* (*s.l.*) cluster and listed as one of 17 neglected tropical diseases by the World Health Organization (WHO) (Larrieu et al., 2019). CE is distributed worldwide, mainly in South America, Eastern Europe, the Middle East, Russia, and Western China, and the prevalence is up to 5% to 10% in highly endemic areas (Agudelo Higuita et al., 2016; Wen et al., 2019). More than 2-3 million cases are estimated worldwide (Craig et al., 2007). According to the WHO, the costs globally committed for CE treatment are more than $3 billion per year (World Health Organization, 2019). China has a high incidence of human CE, accounting for 40% of global DALYs lost worldwide (Budke et al., 2006). Between 2012 and 2016, CE was endemic in 368 counties, with an estimated 166,098 cases of CE in China (Qian and Zhou, 2018).

The early symptoms of CE are not obvious, and most CE patients are already in advanced stages when they seek medical treatment (Wen et al., 2019). This treatment mainly includes surgical and anti-parasitic drug treatments, but surgery is not always possible and may be complicated by postoperative recurrence and secondary infection or biliary leakage (Brunetti et al., 2010). For some patients who are not suitable for surgical treatment, antiparasitic treatment has become the first choice; in this situation, the germinal layer of the metacestode is the main target of the drug. Anti-parasitic treatment is also used before and after operation to prevent a recurrence, with the possibly spilled protoscoleces (PSCs) as the main targets (Kern et al., 2017). Currently, benzimidazoles, mebendazole and more often albendazole (ABZ), are the only drugs that may be used to treat this infection at its metacestode stage, as recommended by WHO, but such drugs are characterized by poor bioavailability, wide inter-individual variations in blood levels, and occurrence of adverse reactions (Horton, 2002; Horton, 2003). At present, we are missing anti-CE drugs that can effectively replace ABZ; in addition, the killing potential of ABZ on PSCs is not optimal, and it is delayed (Horton, 2003). Therefore, the identification of new drug targets and the development of new therapeutic molecules are of great significance for the treatment of CE.

The results of transcriptome and whole-genome sequencing show that after entering the intermediate host, the anabolic ability of *E. granulosus s.l.* is severely degraded, while its ability to absorb nutrients is greatly increased, and complete metabolic pathways such as glycolysis, tricarboxylic acid cycle and pentose phosphate cycles function efficiently (Tsai et al., 2013; Zheng et al., 2013). This suggests that *E. granulosus s.l.* requires nutrients such as glucose from the intermediate host’s environment to meet basic physiological functions such as energy metabolism. If glucose is not transported from the host environment in time or cannot be transported to the parasite, the parasite’s death probably occurs, which is suggested by the presence of specific antibodies to *E. granulosus s.l.* in the serum of patients without lesions in endemic areas, and the observation of abortive forms of disease (Wang et al., 2006). It is considered that interference with glucose metabolism is one of the mechanisms of action of ABZ (Son et al., 2020). However, the precise metabolic chain involved in *E. granulosus s.l.* glucose uptake is unknown.

Glucose transporter 1 (GLUT1) is widely distributed across most cell types and responsible for the transmembrane transport of glucose (Takata et al., 1990). In recent years, GLUT1 has gradually become a research hotspot in the field of metabolic diseases, cancer, and other diseases, and is considered as an important target for drug development (Xiao et al., 2018; Li et al., 2019; Bertrand et al., 2020). Recently, Wei et al. found that inhibiting the human GLUT1 in the erythrocytes could alleviate their injury caused by *Plasmodium* spp. infection (Wei et al., 2018). For some parasites, such as *Trypanosoma* spp. and *Leishmania* spp., hexose transporters have been reported to be involved in the glucose uptake pathway of the parasite (Borst and Fairlamb, 1998; Michels et al., 2006). In addition, GLUT1 of *E. multilocularis* was shown to have relatively high glucose transport activity and to be a crucial participant in the parasite glucose metabolism (Kashiide et al., 2018). WZB117, which binds to the GLUT1 at the exofacial sugar binding site, is a reversible competitive inhibitor of glucose uptake and exchange glucose transport (Ojelabi et al., 2016), and has now been shown to have therapeutic effects in cancer (Liu et al., 2012) and *Plasmodium* infection (Wei et al., 2018). However, we currently do not know whether GLUT1 can be an important drug target in echinococcosis treatment and whether its inhibitor WZB117 has an anti-CE effect.

In our study, we tested whether we could affect *E. granulosus s.l.* glucose uptake by targeting EgGLUT1 at the metacestode stage of *E. granulosus s.l.* We thus cloned a conserved GLUT1 homology gene from *E. granulosus sensu stricto* (*s.s.*), the species most widely responsible for CE cases worldwide, and inhibited EgGLUT1-ss by WZB117 to study its possible impact on the survival of the metacestode *in vitro* and in an experimental animal model.

## Materials and Methods

### Chemicals

WZB117 and albendazole sulfoxide (ABZSO) were obtained from MedChem Express (USA), and dimethyl sulfoxide (DMSO) and ABZ from Sigma (USA).

### Parasite Collection and Culture

Sterile PSCs were obtained aseptically from the cysts of sheep infected with *E. granulosus sensu stricto* (*s.s.*) in the Hualing slaughter market of Urumqi in Xinjiang, PR China. All experiments were conducted on this species. Viable and morphologically intact PSCs were cultured using RPMI-1640 medium (Gibco, USA) (10% fetal bovine serum, 100 U/ml penicillin, and 100 μg/ml streptomycin), and maintained at 37°C under a humidified atmosphere containing 5% CO_2_ (Walker et al., 2004; Wang et al., 2018). For *in vitro* experiments on *E. granulosus s.s.* metacestode, *in vitro* sterile cultures were maintained for 4 months to obtain vesicles with a diameter of approximately 2 mm.

### Experimental Infection of Mice With *E. granulosus s.s.* PSCs

Healthy female Kunming mice (20 ± 2 g of 8 weeks old) were adaptively reared by the Experimental Animal Center of Xinjiang Medical University for one week under controlled laboratory conditions (temperature 20 ± 2°C and 50 ± 5% humidity) (Loos and Cumino, 2015). The mice were inoculated with 2,000 PSCs in 0.2 mL normal saline by intraperitoneal injection. After 6 months, the mice were examined by B-scan ultrasonography; when the diameter of the lesion was more than 0.5 cm, this indicated that the infection was successful.

### WZB117 Treatment *In Vitro*

For a first set of experiments, after PSCs were cultured *in vitro* for one week, their viability was tested by 1% eosin staining (the dead PSCs were stained red with 1% eosin dye, while living PSCs were unstained); to be used in the study, the required percentage of viability required was higher than 90% (Loos et al., 2017). *In vitro* PSCs treatments were performed with 3.125, 6.25, 12.5, 25, 50 and 100 μmol/L WZB117 (dissolved in DMSO). For a second set of experiments, the PSC-derived vesicles were cultured *in vitro* for four months and then cultured in a 6-well plate (25 vesicles/well). *In vitro* vesicle treatments were performed with 3.125, 6.25, 12.5, 25, 50 and 100 μmol/L WZB117 and 15 μmol/L ABZSO. For both control PSCs and vesicles, the culture medium with an identical amount of DMSO (without inhibitor; final concentration, 1%) was used, and the PSCs and vesicles were cultured in an incubator (5% CO_2_, 37°C) for 4 days, and each viability test was repeated three times. The collapse of vesicles, loss of swelling and contraction of germinal layer were used as criteria for evaluating vesicle viability (Elissondo et al., 2007). Viability of PSCs and vesicles was observed under a microscope every 24 h (Fabbri et al., 2016). Each experiment was performed in triplicate and repeated three times.

### Glucose Uptake Assay

Glucose uptake levels were measured using the 2-[*N*-(7-nitrobenz-2-oxa-1,3-diazol-4-yl) amino]-2-deoxy-D-glucose (2-NBDG) (final concentration, 100 μmol/L) assay. Briefly, the PSCs with siRNA sequence or the WZB117-treated PSCs were incubated at 37°C for 48 h. Then, the PSCs were incubated in the darkness with 2-NBDG for 180 min and 60 min at 37°C in 5% CO_2_ humidified atmosphere. The fluorescence intensity was detected at the excitation wavelength of 466 nm and emission wavelength of 540 nm by the fluorescence marker.

### WZB117 Treatment *In Vivo*

For *in vivo* experiments, ABZ and WZB117 was dissolved in PBS/DMSO (1:1, v/v) (Liu et al., 2012). All *E. granulosus s.s.-*infected mice (n = 45) judged appropriate at the 6^th^ month after infection (see above) were randomly divided into five experimental groups (9 mice per group): control group (receiving injections of the PBS/DMSO solvent, 1:1, v/v), ABZ (50 mg/kg/day) group (Wen et al., 1996) and WZB117 (5, 10, 20 mg/kg/day) groups (Wei et al., 2018). After 28 days of continuous intraperitoneal injection (dosage: 0.1 mL/10 g per mouse), animals were sacrificed by cervical dislocation (Loos et al., 2017). At necropsy, the peritoneal cavity was opened, the number of cysts was counted, cyst weights and diameters were measured. The efficacy of treatments was calculated as follows: 100 × {(average cyst weight in the control group) - (average cyst weight in the treatment group)}/(average cyst weight in the control group) (Loos et al., 2017).

### Ultrastructure Observations of WZB117 Treated PSCs, Vesicles, and Cysts

After *in vitro* and *in vivo* drug treatment, the PSCs, vesicles, or cysts were fixed with 4% glutaraldehyde for 24 h (Xin et al., 2019). The samples were processed for SEM using a LEO1430VP (LEO company, Germany) microscopy and TEM using a JEM1230 (JEOL company, Japan) microscope, as previously described.

### Glucose and ATP Content Measurements

To determine the glucose and ATP content of cysts in treated mice, the cysts were washed three times with precooled PBS and added with 100 μL of 20 mM Tris buffer (Thermo, USA). The cyst wall was homogenized in a homogenizer for 2~4 minutes and boiled in a water bath for 5 minutes before centrifugation (at 15,800 g at 4°C for 30 min) (You et al., 2009). The cyst fluid was processed by gradient dilution according to the instructions in the kit. Glucose Colorimetric Assay Kit (Cayman, USA) was used to determine glucose contents. The ATP content was detected by the ATP Detection Assay Kit (Cayman, USA). Experimental groups include at least 5 mice. Each experiment was repeated three times.

### EgGLUT1-ss Cloning

Total RNA was extracted from *E. granulosus s.s.* PSCs by Mini BEST Universal RNA Extraction Kit (Takara, Japan). Me Script II 1^st^ Strand cDNA Synthesis Kit (Takara, Japan) was reverse transcriptionally synthesized for 1^st^ cDNA. The reaction conditions were: incubation at 42°C for 60 min, heating at 95°C for 5 min, termination of the reaction, and storage at -20°C (Cumino et al., 2010). We used the 1^st^ cDNA synthesized by reverse transcription as a template and used the Premix Ex Taq™ Hot Start Version (Takara, Japan) to amplify the full-length sequence of the EgGLUT1-ss gene; primers for the target genes included: EgGLUT1-ss (forward primer 5’- ATGGTTAACTTTCACTACGT-3’ and reverse primer 5’-CTAAAATCTGACCTTATCG-3’). The PCR amplification conditions were: pre-denaturation at 94°C for 5 min; denaturation at 98°C for 30 s; annealing at 45°C for 30 s; extension at 72°C for 90 s, for a total of 34 cycles. The reaction was terminated after 10 min extension at 72°C; 1% agarose gel was used to determine whether the gene band location was found where expected. PCR products of EgGLUT1-ss gene were recovered from Agarose Gel with Agarose Gel DNA Extraction Kit (Takara, Japan), and the amplified fragments were cloned into pMD19-T vector with Mighty ta-cloning Reagent Set for Prime STAR (Takara, Japan), and verified by sequencing. The gene was named as EgGLUT1-ss (GenBank accession number: QTT61014.1).

### Bioinformatics Analysis and Construction of the Phylogenetic Tree of GLUT1

The amino acid sequences of the homologous genes of GLUT1 in *E. multilocularis* (GenBank ID: CDS42031.1), *Hymenolepis microstoma* (GenBank ID: CDS25463.1), *Caenorhabditis elegans* (GenBank ID: NP_493981.1), *Drosophila melanogaster* (GenBank ID: NP_001097467.1), *Labrus bergylta* (GenBank ID: XP_020502389.1), *Astyanax mexicanus* (GenBank ID: XP_007258287.2), *Xiphophorus maculatus* (GenBank ID: XP_023187106.1), *Aplysia californica* (GenBank ID: XP_012944940.1), *Biomphalaria glabrata* (GenBank ID: XP_013087453.1), *Crassostrea gigas* (GenBank ID: XP_019925400.1), *Danio rerio* (GenBank ID: NP_001034897.1), *Mus musculus* (GenBank ID: XP_006502971.1) and *Homo sapiens* (GenBank ID: NP_006507.2) were obtained from GenBank. The post-translational modification sites were predicted using the MotifScan software (Rai et al., 2014). The transmembrane region was predicted using TMHMM (Gulyaeva et al., 2020). The conserved domain was predicted using GenBank tools. The multiple sequence alignment was analyzed by DNAMAN (Version 7.0.2.176) (Kaukonen et al., 2000). The phylogenetic tree was constructed by MEGA (Version 10.0.5) (Hall, 2013).

### EgGLUT1-ss-siRNA Interference

According to the cloned EgGLUT1-ss Gene sequence (GenBank accession number: QTT61014.1), siRNA interference sequences of three EgGLUT1-ss genes were designed using Gene Pharma siRNA Designer 3.0 software. Experimental groups were: siRNA-386-treated group, siRNA-578-treated group, siRNA-723-treated group, negative control group (NC, transfection independent interference sequence) and untreated group. The three siRNA sequences were transfected into *in vitro* cultured PSCs separately. The targeting sequence of each siRNA in EgGLUT1-ss cDNA is summarized in **Table 1**. The EgGLUT1-ss-siRNA interference assay was carried out as previously described (Mizukami et al., 2010). Briefly, electroporation was performed at 125 V using a pulse. For electroporation, 200 μL electroporation buffer (5 mM magnesium chloride, 200 mM glucose, 20 mM Tris, 2 mM 2-Hydroxy-1-ethanethiol, pH adjusted to 7.4 with HCl) containing approximately 4,000 PSCs was placed in a 4-mm cuvette, and the final concentration of siRNA interference sequence was 5 μM. After electroporation, the shock cup was placed in a 37°C incubator for 30 min and then transferred to a 6-well plate with 2 mL mixed medium for 48 h. After washing with PBS, PSCs in each group were stained with 1% eosin for 5 min (the dead PSCs were stained red with 1% eosin dye, while living PSCs were unstained), respectively. The PSCs viability was evaluated by counting living and dead PSCs using a microscope.

**Table 1:** Design of the potential three siRNA sequences targeting EgGLUT1-ss. Gene.

siRNA ID	Sense sequences	Antisense sequences
siRNA-386	GGAUUGAACCUGGUCCGAUTT	AUCGGACCAGGUUCAAUCCTT
siRNA-578	CCACGGUGAUGACAACAAUTT	AUUGUUGUCAUCACCGUGGTT
siRNA-723	GGCAACAAAGAGAGCCAAATT	UUUGGCUCUCUUUGUUGCCTT

### Quantitative Real-Time Polymerase Chain Reaction (qRT-PCR) Detection

Total RNA was extracted from PSCs and vesicles by Mini BEST Universal RNA Extraction Kit (Takara, Japan), as previously described (Li et al., 2004). RNA was converted to cDNA with the PrimeScript™ RT reagent Kit Prime Script TMRT reagent Kit (Takara, Japan), and the cDNA was analyzed by qRT-PCR using TB Green^®^ Premix Ex Taq™ II (Takara, Japan). The primer sequences are shown in **Table 2**. All samples were run in triplicate using the following cycle parameters: 95°C for 30 s; 40 cycles at 95°C for 5 s and 55°C for 30 s. All data were used for standard curve analysis.

**Table 2:** EgGLUT1-ss Gene primer sequences.

Gene	Primer	Sequence 5’-3’
EgGLUT1-ss	Fw	5´- TTCTTCTTATAGGCGGTCTC -3´
	Rv	5´- AGGTGATGGCAAGGTAGA -3´
β-actin	Fw	5´-GCGATGTATGTAGCTATCCAGGCAGTGCTCTCGCT-3´
	Rv	5´-CAATCCAGACAGAGTATTTGCGTTCCGGAGGA-3´

### Docking Study

Through the Pubchem website (https://pubchem.ncbi.nlm.nih.gov/) to retrieve the 3D structure of WZB117, we adopted the Autodock software (version 4.2.6) to hydrogenation processing, calculating charge EgGLUT1-ss protein, and setting the receptor proteins docking lattice parameter. We used AutoDock Vina (version 1.1.2) to dock EgGLUT1-ss and WZB117 and obtained 20 conformations. EgGLUT1-ss and WZB117 were used to chart the binding sites and amino acid residues and PyMOL (version 2.4.0) was used as a 3D diagram to show the interaction between receptor proteins and ligand small molecules.

### *In Vitro* Toxicity Assessment of WZB117 on Primary Mouse Hepatocytes

Primary hepatocytes were isolated from 10 - 12 weeks old female C57BL/6 mice and the isolation was performed as previously described (Liu et al., 2021). Isolated primary mouse hepatocytes were inoculated at a density of 1 × 10^4^ cells/0.32 cm^2^ growth area per well in rat tail collagen type I-coated 96-well plates for 4 h at 37°C, 5% CO_2_, and incubated in a growth medium [William’s E (Gibco, USA), 10% fetal bovine serum, 0.01% dexamethasone, 0.25% insulin and 1% of 100 U/ml penicillin/100 μg/ml streptomycin] for further experiments. Cell Counting Kit-8 (CCK-8) (Bioss, China) assay is used to detect the viability of primary mouse hepatocytes in each group. Briefly, primary mouse hepatocytes were cultured at 37°C and 5% CO_2_ for 4 h. The medium was replaced with medium (without serum) and 1% DMSO was used as the control group, while WZB117 was added to the medium at final concentrations of 3.125, 6.25, 12.5, 25, 50 and 100 μmol/L to continue the culture for 48 h. Then 10 μL of CCK-8 solution was added to each mixed well, and after 2 h of continuous incubation, zeroing was completed using blank control wells, and the optical density of each well was measured at 450 nm using a microplate reader (Thermo Fisher Scientific, USA).

### Statistical Analysis

All analyses were performed using IBM SPSS Statistics 20 software. The chi-square test was used at each time point to assess the effect of *E. granulosus s.s.* PSCs and vesicles viability in the treatment and control groups. The one-way analysis of variance (ANOVA) was used to analyze the effect of siRNA interference and WZB117 on the glucose metabolism of *E. granulosus s.s.* PSCs. The one-way ANOVA and nonparametric test of Kruskal-Wallis test were used to assess the efficacy of WZB117 treatment in *E. granulosus s.s.*-infected female Kunming mice. All data were expressed as (mean ± S.D.), and *P* values are indicated in each assay (*P < 0.05, **P < 0.01). For all analyses *P* < 0.05 was considered statistically significant.

### Linguistic Statement

The terminology proposed by the World Association of Echinococcosis was followed all along the text of the publication. Especially, we followed the distinction between ‘vesicles’, obtained *in vitro*, and ‘cysts’ obtained from *in vivo* experiments, and between *E. granulosus sensu lato* for the cluster of species previously known as ‘*E. granulosus’* and ‘*E. granulosus sensu stricto’* for the species used in our experiments (Vuitton et al., 2020).

## Results

### Cloning, and Physiological and Biochemical Characterization of EgGLUT1-ss

**Figure 1:**
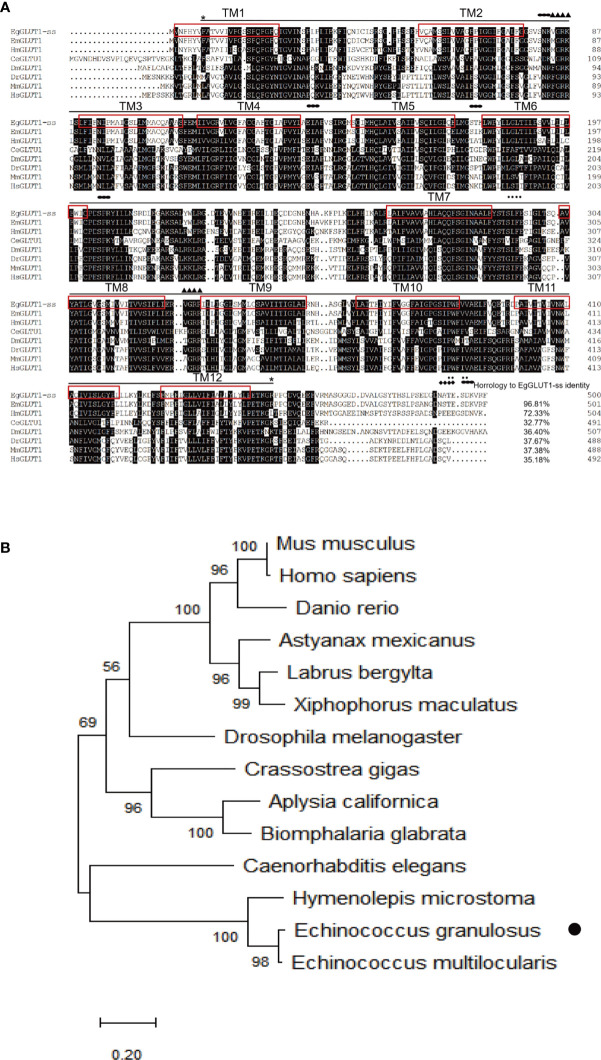
EgGLUT1-ss bioinformatics analysis. **(A)** Multiple-sequence alignments of EgGLUT1-ss from *E granulosus s.s.* (GenBank accession number: QTT61014.1); *Echinococcus multilocularis* (GenBank accession number CDS42031.1); *Hymenolepis microstoma* (GenBank accession number CDS25463.1); *Caenorhabditis elegans* (GenBank accession number NP_493981.1); *Drosophila melanogaster* (GenBank accession number NP_001097467.1); *Danio rerio* (GenBank accession number NP_001034897.1); *Mus musculus* (GenBank accession number XP_006502971.1); and *Homo sapiens* (GenBank accession number NP_006507.2). The horizontal line between the two * represents the MFS super family; (▲) denotes the amidation site; (◆) denotes the glycosylation site; (·) denotes Casein kinase II phosphorylation site; (●) denotes protein kinase C phosphorylation site. The red box represents the transmembrane (TM) region. **(B)** Phylogenetic tree constructed using the neighbor-joining method to compare the relationship between EgGLUT1-ss and GLUT1 from other species. The numbers above the branches refer to bootstrap values. The species for sequences included in the phylogenetic analysis are shown behind the branches. EgGLUT1-ss from *E granulosus s.s.* is indicated by •.

To assess whether GLUT1 gene was present in *E. granulosus s.s*, we successfully cloned a conserved GLUT1 homologous gene from *E. granulosus s.s.*, named EgGLUT1-ss (GenBank accession number: QTT61014.1) (**Supplementary Figure 1**), which encodes 500 amino acids (**Figure 1A**). Bioinformatics analysis showed that EgGLUT1-ss had one major facilitator superfamily (MFS) domain and 12 transmembrane regions, which are a typical feature of the GLUT gene (**Figure 1A**). This special structure plays an important role in the transmembrane transport of glucose (Hruz and Mueckler, 2001). In addition, we also found that EgGLUT1-ss contained two amidation sites, one glycosylation site, one cAMP- and cGMP-dependent protein kinase phosphorylation site, two casein kinase II phosphorylation sites, five myristoylation sites, and five protein kinase C phosphorylation sites (**Figure 1A**). Whole sequence alignment analysis showed that the highest overall homologies on the amino acid sequence level could be detected between EgGLUT1-ss and the GLUT1 of *E. multilocularis* (96.81% identical). When EgGLUT1-ss sequence was compared with the sequences of GLUT1 in other species, percentages of homology were between 32.77% to 72.33% (**Figure 1A**). Phylogenetic analysis revealed that the amino acid sequence of EgGLUT1-ss was generally closely related to parasite species GLUT, especially helminths, and was more distantly related to mammalian species such as human and mouse GLUT (**Figure 1B**).

### EgGLUT1-ss Knockdown Reduces the Viability and Glucose Uptake of *E. granulosus s.s.* PSCs

**Figure 2:**
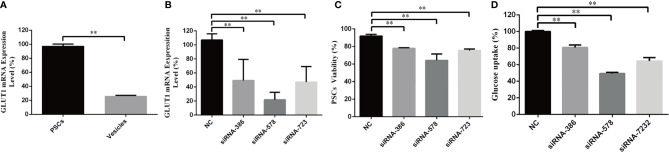
EgGLUT1-ss Knockdown reduces the viability and glucose uptake of PSCs. **(A)** EgGLUT1-ss mRNA expression in PSCs and vesicles. **(B)** EgGLUT1-ss mRNA expression at 48 h after siRNA-386, siRNA-578 and siRNA-723 interference. **(C)** PSCs viability at 48 h after siRNA-386, siRNA-578 and siRNA-723 interference. **(D)** Glucose uptake of PSCs at 48 h after siRNA-386, siRNA-578 and siRNA-723 interference. ** Show a statistically significant difference between the siRNA interference group and the NC group at the indicated times (**P < 0.01) (chi-square test and one-way ANOVA). Data are the mean ± S.D. of three independent experiments. NC, negative control group (transfection by independent interference sequence).

To investigate EgGLUT1 function in *E. granulosus s.l.*, we examined the EgGLUT1-ss expression in PSCs and vesicles of *E. granulosus s.s.* We found EgGLUT1-ss was expressed in both PSCs and vesicles, and that EgGLUT1-ss expression level in PSCs was 3.5 times higher than that of vesicles (*P* < 0.01) (**Figure 2A**).

To determine the functional relevance of EgGLUT1-ss in PSCs, we designed three siRNA sequences (siRNA-386, siRNA-578 and siRNA-723) targeting EgGLUT1-ss. The three siRNA compositions treatment led to a significant reduction in EgGLUT1-ss-mRNA expression in PSCs compared with the NC group (siRNA-386, *P* < 0.01; siRNA-578, *P* < 0.01; siRNA-723, *P* < 0.01) (**Figure 2B**). To detect the viability of PSCs, the number of PSCs was calculated after 1% eosin staining (the dead PSCs were stained red with 1% eosin dye, while living PSCs were unstained), and the results showed that the viability of PSCs was significantly reduced in all three siRNA intervention groups compared with the NC group (siRNA-386, *P* < 0.01; siRNA-578, *P* < 0.01; siRNA-723, *P* < 0.01) (**Figure 2C**). To examine the influence of EgGLUT1-ss knockdown on the glucose uptake of PSCs, three siRNA compositions were transfected into PSCs, and 2 days after transfection, compared with the NC group, 2-NBDG uptake by PSCs decreased significantly (siRNA-386, *P* < 0.01; siRNA-578, *P* < 0.01; siRNA-723, *P* < 0.01) (**Figure 2D**).

### WZB117 Inhibits Glucose Metabolism and Reduces the Viability of *E. granulosus s.s.* PSCs *In Vitro*

**Figure 3:**
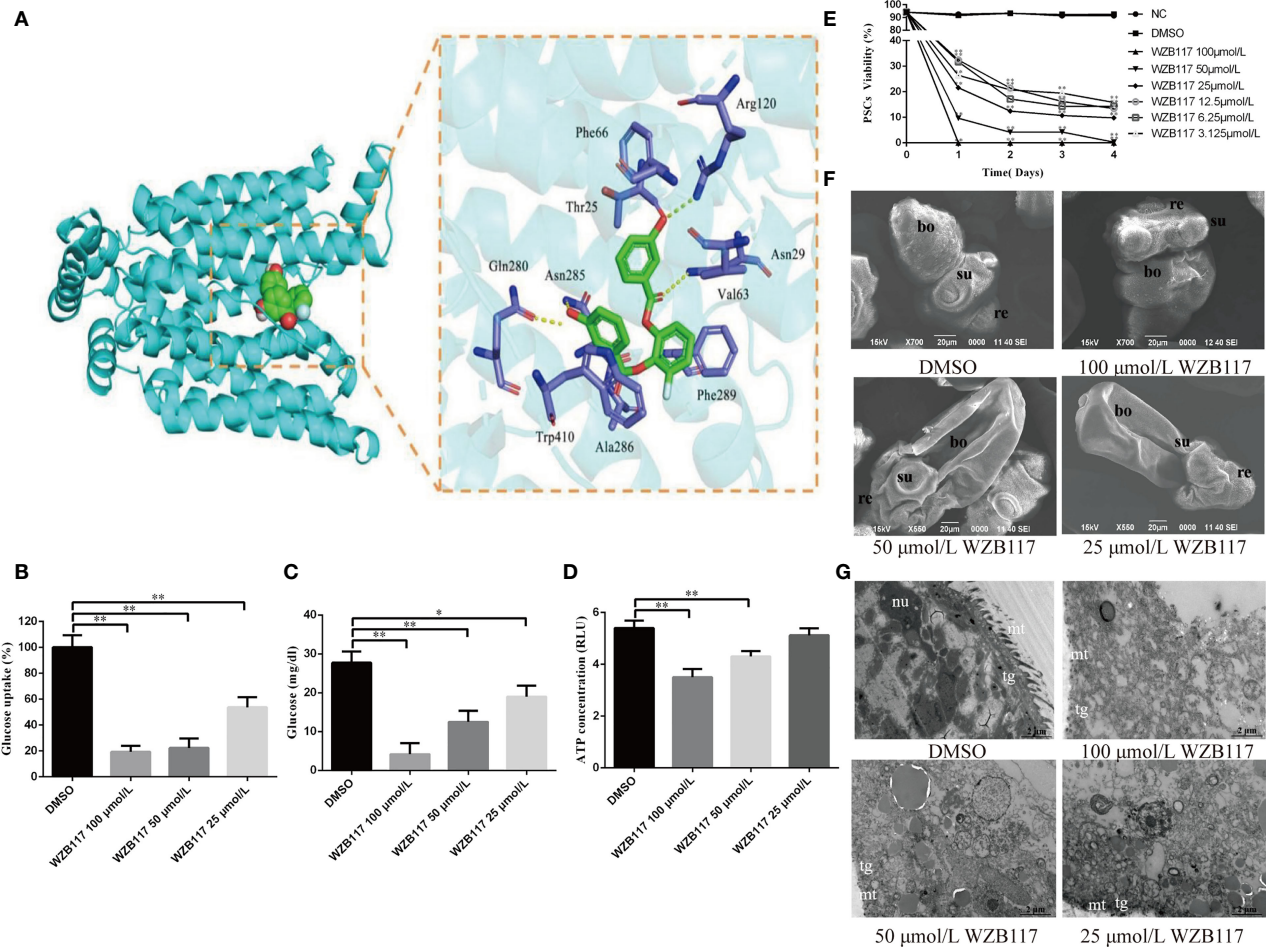
WZB117 inhibits glucose metabolism and reduces the viability of PSCs *in vitro*. ***(*A*)*** Docked structure and interactions of WZB117 binding to EgGLUT1-ss. The image on the left shows that WZB11 binds to the central channel region of EgGLUT1-ss. The image on the right shows the detailed interactions (formation of 4 hydrogen bonds) between WZB117 and amino acid residues of EgGLUT1-ss. **(B)** Glucose uptake of PSCs after WZB117 incubation for 60 min. **(C)** Glucose content of PSCs after WZB117 incubation for 48 h. **(D)** ATP content of PSCs after WZB117 incubation for 48 h. **(E)** PSCs viability after WZB117 intervention for 4 days. Each point represents the mean percentage of viable PSCs from three different experiments. * and ** show a statistically significant difference between the treatment group and the DMSO group at the indicated times (*P < 0.05, **P < 0.01) (chi-square test and one-way ANOVA). Data are the mean ± S.D. of three independent experiments. **(F)** Representative SEM images of PSCs after WZB117 incubation for 4 days. PSCs incubated in culture medium containing DMSO served as a control. **(G)** Representative TEM images of PSCs after WZB117 intervention for 4 days. PSCs incubated in culture medium containing DMSO served as a control. NC, negative control group; DMSO, control group; re, rostellum; su, suckers; bo, body; mt, microtriches; nu, nucleus; tg, tegument.

By docking predictions, we found that WZB117 had the potential ability to combine with EgGLUT1-ss. The binding of WZB117 to EgGLUT1-ss involved four hydrogen bonds, with Arg120, Asn29, Asn285 and Gln280. Amino acid residues Phe66, Thr25, Val63, Phe289, Ala286, and Trp410 interacted with the WZB117 molecule through hydrophobic bonds (**Figure 3A**). After 60 min of incubation with WZB117 *in vitro*, the glucose uptake level of PSCs was significantly decreased (*P* < 0.01) (**Figure 3B**). After 48 h incubation with 25 (*P* < 0.05), 50 (*P* < 0.01), and 100 (*P* < 0.01) μmol/L WZB117 *in vitro*, the glucose content of PSCs was significantly decreased compared with the DMSO group (**Figure 3C**). In parallel, compared with the NC group, the ATP content was decreased (**Figure 3D**) when the PSCs were treated with 50 (*P* < 0.01) and 100 (*P* < 0.01) μmol/L WZB117.

To investigate the effect of WZB117 on the viability of *E. granulosus s.s.* larval stages *in vitro*, we analyzed the survival rate of PSCs exposed to various concentrations of WZB117. As shown in **Figure 3E**, compared to the DMSO group, 4 days-WZB117 exposure led to a dose-dependent decrease in the viability of PSCs in cultures (*P* < 0.01). After exposure to 100 μmol/L WZB117 *in vitro* for one day, all the PSCs died. Scanning electronic microscopy (SEM) showed that, compared to the DMSO group, WZB117-treated PSCs exhibited incomplete rostellum structure, absence of hooks, collapsed suckers and various damages to the other PSC structures, as shown in **Figure 3F**. Transmission electronic microscopy (TEM) showed that there were significant changes in the ultrastructure of WZB117-treated PSCs: the microtriches disappeared, the nuclei were ruptured, associated with disappearance of the nucleoli, and vacuoles appeared in the cytoplasm. (**Figure 3G**).

### WZB117 Reduces the Viability of *E. granulosus s.s.* Vesicles *In Vitro*

**Figure 4:**
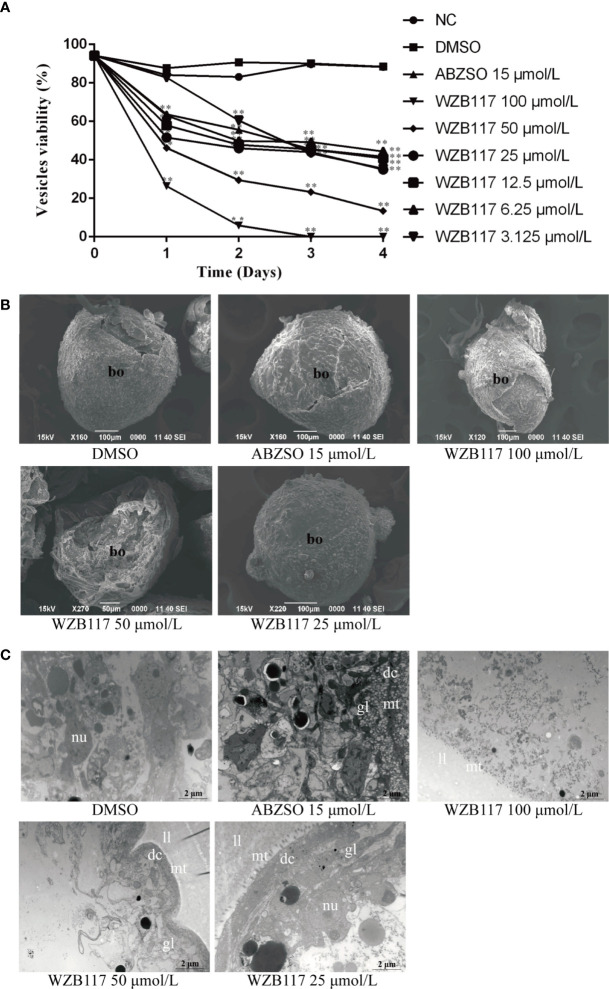
WZB117 reduces the viability of *E granulosus s.s.* vesicles *in vitro*. **(A)** Vesicle viability of *E granulosus s.s.* after WZB117 incubation for 4 days. Each point represents the mean percentage of viable vesicles from three different experiments. * and ** show a statistically significant difference between the treatment group and the DMSO group at the indicated times (*P < 0.05, **P < 0.01) (chi-square test). NC: negative control group; DMSO: control group; albendazole sulfoxide (ABZSO) at 15 μmol/L was used as a reference anti-infective agent. Data are the mean ± S.D. of three independent experiments. **(B)** Representative SEM images of vesicles after WZB117 incubation for 4 days. Vesicles incubated in culture medium containing DMSO served as a control. **(C)** Representative TEM images of vesicles after WZB117 incubation for 4 days. Vesicles incubated in culture medium containing DMSO served as a control. bo, body; gl, germinal layer; ll, laminated layer; mt, microtriches; nu, nucleus.

Cysts are the lesions produced by *E. granulosus s.l.* in the intermediate host and their development is related to the pathogenicity of this helminth (Wen et al., 2019). Therefore, we further explored the effect of WZB117 on *in vitro*-cultured vesicles exposed to increasing concentrations of WZB117. Compared with the DMSO group, the viability of the vesicles was significantly decreased when they were exposed to various concentrations of WZB117 for 4 days (*P* < 0.01); it was decreased by 100% on day 3 when exposed to 100 μmol/L WZB117 (**Figure 4A**). These data and morphological changes indicate that WZB117 had a significant effect on the reduction of the viability of *in vitro-*developed *E. granulosus s.s.* vesicles. Compared with the DMSO group, the SEM features of the vesicles in the WZB117 group changed significantly: the germinal layer was condensed, and the vesicles collapsed. The degree of collapse was positively correlated with the dose of WZB1117 (**Figure 4B**). TEM images obtained from the WZB117-treated vesicles showed that the structures of germinal layer and laminated layer were damaged, lipid droplet appeared, the microtriches were reduced, nucleoli disappeared, the heterochromatin edges were clustered, and the cortical matrix was fuzzy (**Figure 4C**).

### WZB117 Treatment Affects Glucose/ATP Levels and Effectively Prevents the Growth of Cysts in *E. granulosus s.s.-*Infected Mice

**Figure 5:**
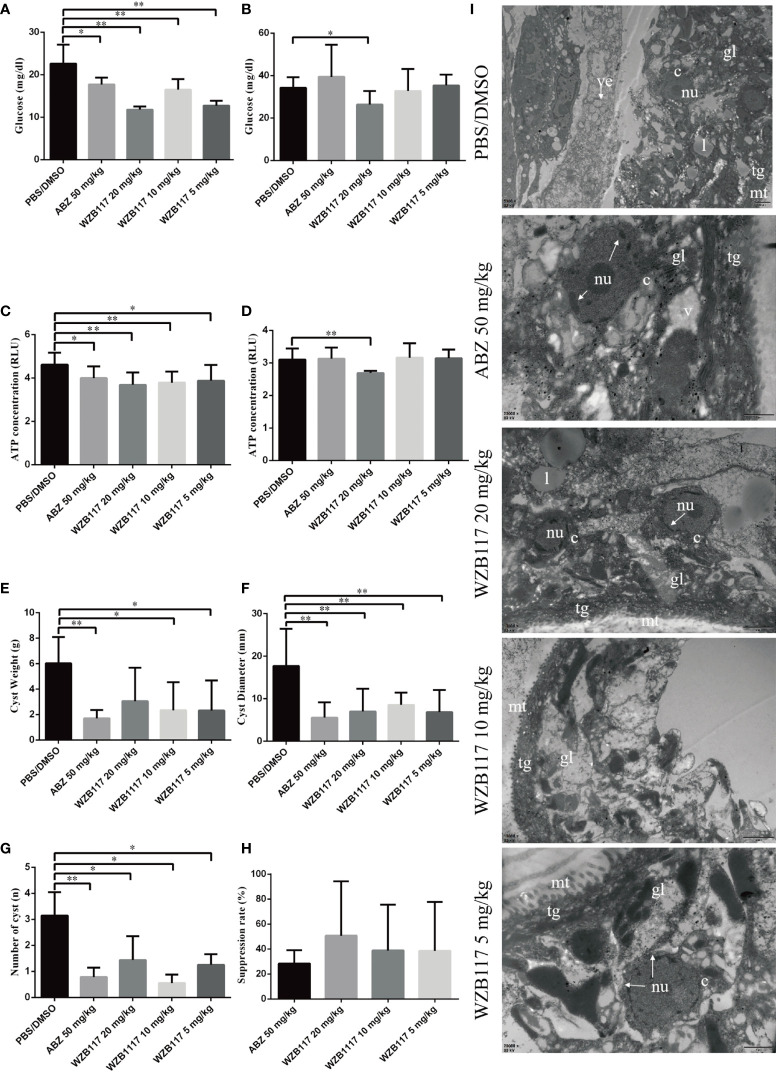
WZB117 treatment effectively inhibits the growth of cysts and reduces glucose and ATP levels in *E granulosus s.s.-*infected mice. **(A)** Glucose content of cyst tissue in *E granulosus s.s.*-infected mice after WZB117 treatment for 28 days. **(B)** Glucose content in the cyst fluid after WZB117 treatment for 28 days. **(C)** ATP content in the cyst tissue. **(D)** ATP content in the cyst fluid. **(E)** Cyst weight in *E granulosus s.s.*-infected mice. **(F)** Cyst number in *E granulosus s.s.*-infected mice. **(G)** Cyst diameter in *E granulosus s.s.*-infected mice. * and ** show a statistically significant difference between the treatment group and the PBS/DMSO group at the indicated times (*P < 0.05, **P < 0.01) (one-way ANOVA and nonparametric test of Kruskal-Wallis test) (n ≧ 5). **(H)** Efficacy of 28-days treatments in *E granulosus s.s.*-infected mice. **(I)** Representative TEM images of cysts in *E granulosus s.s.*-infected mice. PBS/DMSO (1:1, v/v): control group; albendazole (ABZ) at 50 mg/kg was used as a reference anti-infective agent. gl, germinal layer; ll, laminated layer; tg, tegument; l, lipid droplet; mt, microtriches; nu, nucleus; c, cell; arrow, broken nucleus.

We measured the glucose and ATP content of the cysts *in vivo*. Compared with the PBS/DMSO group, there was a marked reduction in glucose (5 mg/kg WZB117-treated group, *P* < 0.01; 10 mg/kg WZB117-treated group, *P* < 0.01; 20 mg/kg WZB117-treated group, *P* < 0.01) and ATP (5 mg/kg WZB117-treated group, *P* < 0.05; 10 mg/kg WZB117-treated group, *P* < 0.01; 20 mg/kg WZB117-treated group, *P* < 0.01) levels in the cyst tissue after WZB117 treatment (**Figures 5A, C**). A significant reduction of glucose (*P* < 0.05) and ATP (*P* < 0.01) levels in the cyst fluid was observed for the 20 mg/kg dose of WZB117 (**Figures 5B, D**).

After 6 months of WZB117 treatment, the weight of cysts recovered from 5mg/kg and 10 mg/kg WZB117-treated mice was significantly lower than that recovered from the PBS/DMSO group (*P* < 0.05) (**Figure 5E**). Additionally, the number (*P* < 0.01) and diameter (*P* < 0.05) of cysts in WZB117-treated mice were lower than those of the PBS/DMSO group (**Figures 5F, G**). Overall, the ‘therapeutic’ effect of 10 mg/kg WZB117 was comparable to that of ABZ (**Figure 5H**).

In the PBS/DMSO group (**Figure 5I**), the cysts had no significant structural abnormalities when observed with TEM. However, the structure of cysts removed from the treated groups was damaged at different degrees (**Figure 5I**). Compared with the PBS/DMSO group, cysts from WZB117-treated mice had loose germinal layers, the microtriches were shortened and reduced dramatically in length, the tegument was thinned, the structure of the laminated layer was destroyed, and the nuclei were condensed.

For a preliminary assessment of the safety of WZB117 in the treatment of CE in experimental hosts, we first evaluated the toxicity of WZB117 on primary mouse hepatocytes by the CCK-8 assay. cells were treated with WZB117 at concentration series (3.125, 6.25, 12.5, 25, 50 and 100 μmol/L) for 48 h (**Supplementary Figure 2**). The results showed that the primary mouse hepatocyte activity in the 100 μmol/L WZB117 group was 95.98 ± 10.38% compared to the 1% DMSO group (*P* > 0.05), and the primary mouse hepatocyte viability in the other treatment groups was also not significantly different from the 1% DMSO group (*P* > 0.05), indicating that WZB117 was not significantly toxic to primary mouse hepatocytes at the effective dose. In the *in vivo* experiments, we measured the usual indices of adverse effects. There were no differences in body weight between treated and control mice. The aspartate transaminase (AST) level was significantly lower in the 5 mg/kg WZB117-treated group than in the PBS/DMSO group (*P* < 0.05) (**Supplementary Figure 3A**) and there were no significant differences in alanine aminotransferase (ALT) and alkaline phosphatase (ALP) levels, the most accurate markers of liver damage (**Supplementary Figures 3B, C**). The 5 mg/kg WZB117-treated group had lower blood urea levels than the PBS/DMSO group (*P* < 0.05) (**Supplementary Figure 3D**) but all observed values remained in the physiological range and creatinine levels were similar in all WZB117-treated groups and in the PBS/DMSO group (**Supplementary Figure 3E**). We measured blood glucose in mice on days 0, 7, 14, 21 and 28 prior to administration and showed no difference in blood glucose levels between WZB117-treated mice and PBS/DMSO control mice (**Supplementary Figure 4**). Overall, WZB117, at the dosages used in our study did not cause significant toxicity in mice.

## Discussion

The energy-associated metabolic pathways are essential for the survival of parasites and their adaptation to their successive hosts (Vinaud and Ambrosio, 2020) and glucose metabolism is the main energy source (Tsai et al., 2013). Glucose metabolism pathways, especially glycolysis, had been evidenced as a target for anti-echinococcosis drugs (Xin et al., 2019). However, the upstream pathway of glucose metabolism in *E. granulosus s.l.*, which is involved in glucose uptake from the host, was still elusive. In this study, we identified EgGLUT1-ss as a crucial protein involved in glucose uptake by *E. granulosus s.s.* metacestode and demonstrated that functional EgGLUT1-ss was essential for energy-sourcing and survival in the intermediate host of the parasite. We also characterized EgGLUT1-ss as an important drug target against this larval stage and its inhibitor WZB117 as a candidate anti-CE drug.

Glucose metabolism pathway not only produces ATP for the physiological functions of eucaryote cells, but also provides an important source of carbon to support the biosynthesis of nucleotides and non-essential amino acids (Zhu and Thompson, 2019). *E. granulosus s.l.* produces ATP for its own growth by both aerobic and anaerobic carbohydrate metabolism pathways (Cui et al., 2013). Tsai et al. (2013) and Zheng et al. (2013) found that *E. granulosus s.l.* has complete glucose metabolism pathways, including glycolysis, the tricarboxylic acid cycle, and the pentose phosphate pathway. (Wu et al., 2018) showed that the glycolytic enzyme, triosephosphate isomerase, a drug target for the control of schistosomes (Chen and Wen, 2011) and of *Plasmodium* spp. (Velanker et al., 1997), was also involved in the growth and development of *E. granulosus s.l.* In addition, other glycolytic enzymes, such as 6-phosphofructokinase 1 and pyruvate kinase, have been identified in *E. granulosus s.l.* (Pan et al., 2014), and Hemer et al. (2014) showed that the insulin receptor pathway could regulate *E. multilocularis* glucose metabolism. In this study, we found that suppressing the function of EgGLUT1-ss, a member of the glucose transporter 1 family that we cloned from *E. granulosus s.s.*, the species most frequently responsible for CE worldwide, not only inhibited glucose uptake and ATP content by *E. granulosus s.s.*, but also affected the viability of both vesicles and PSCs of *E. granulosus s.s. in vitro* and cysts developed *in vivo*. Glucose, a polar and hydrophilic molecule, cannot pass through hydrophobic cell membranes; GLUT1, which is embedded in the cell membrane, carries out the function of glucose transport to provide glucose supply (Baldwin et al., 1995; Tetaud et al., 1997; Macheda et al., 2005). GLUT1-like transporters have been identified in trypanosomes such as *T. brucei, and T. cruzi*, and in *Leishmania* spp. (Rodriguez-Contreras and Landfear, 2014), as well as in *E. multilocularis*, another species of the *Echinococcus* genus; in that species, EmGLUT1 has a high glucose transport activity and likely plays an important role in glucose uptake from its host at the larval stage (Kashiide et al., 2018). Like *E. multilocularis*, all species within the *E. granulosus s.l.* cluster are also dependent on its intermediate host-derived glucose; we may thus suggest that EgGLUT1, a crucial upstream member of glucose metabolism pathways acting as a switch for glucose to enter the parasite, serves as an energy-provider from the host’s glucose to the *E. granulosus s.l.* metacestode, and is thus essential for its growth and survival.

The systemic anti-parasitic treatment of CE currently relies on the continuous administration of either of two benzimidazole carbamates, ABZ and mebendazole, which are the only drugs clinically efficient to interrupt the larval growth of *E. granulosus s.l.* Both drugs interfere with glucose metabolism: their mechanism of action has been associated with a marked inhibition of pyruvate kinase, phosphoenolpyruvate carboxykinase and ATPase (Hernández-Luis et al., 2010). In addition, a few bioenergetic modulators have shown significant inhibition of parasite viability in preclinical *in vivo* and *in vitro* experiments. As examples, 3-bromopyruvate which blocks glucose entry into the glycolysis pathway by inhibiting hexokinase (HK) (Ko et al., 2001) has been shown to inhibit *Echinococcus* spp. viability *in vitro* and *in vivo* (Xin et al., 2019); tacrolimus, a rapamycin-target protein inhibitor, exerts anti-CE effects *in vivo* and *in vitro* and affects the glucose metabolism of cysts *in vivo* (Muhedier et al., 2020); and metformin can reduce the larval viability of *E. granulosus s.l.* by inhibiting its glucose metabolism pathway (Loos and Cumino, 2015). However, none of these compounds have succeeded in reaching the pre-clinical stage of drug development for CE.

Synthetic GLUT1 inhibitors, such as BAY-876, WZB117 and STF-31, are still in the pre-clinical research stage, but there are obvious experimental data showing that these inhibitors have real therapeutic potential through the inhibition of glucose uptake (Matsumoto et al., 2016; Ma et al., 2018; Peng et al., 2019). Previous studies have reported that WZB117 significantly inhibits the proliferation of cancer cells by reducing the transporter function of GLUT1 (Vander Heiden et al., 2009; Koch et al., 2015; Chen et al., 2017) which was overexpressed in cancer cells (Ganapathy et al., 2009) maybe through the reduction of ATP and glycolytic enzyme levels. Resveratrol, which has a GLUT1 inhibitory effect, has been endorsed by the FDA for the treatment of spinocerebellar ataxia (Meng et al., 2019). WZB117 was also shown to inhibit the growth of blood-stage *P. berghei* and reduce glucose uptake in the red blood cells by breaking redox balance (Wei et al., 2018). In our study, we found that GLUT1 inhibitor WZB117 significantly reduced the viability of *E. granulosus s.s.* Our *in vitro* results showed that WZB117 inhibited the function of EgGLUT1-ss, leading to a reduction in glucose and ATP levels, which ultimately led to the death of both the PSCs and metacestode vesicles *in vitro*. In our *in vivo* experiments, we found that WZB117 achieved the same therapeutic results as ABZ, at a lower dose. The lower levels of glucose content in the cyst wall and the cyst fluid we found in the mice treated by WZB117 may suggest that WZB117 could be slightly more effective than ABZ; which may be due to the different targets of the two drugs, inhibition of tubulin for ABZ with only secondary consequences on glucose metabolism (Tejada et al., 1987), on one hand, and direct impairment of EgGLUT1-ss function in glucose uptake and transport for WZB117 on the other hand. The lower anti-CE effect of ABZ may also be related to its poor solubility, thus low cell availability, a well-known disadvantage for its clinical use in the treatment of echinococcosis (Horton, 2002; Horton, 2003; Brunetti et al., 2010; Kern et al., 2017). The dual and rapid effect of WZB117 on PSCs and the germinal layer of the metacestode is a supplementary advantage of the GLUT1 inhibitor for its use in CE in the peri-operative period to prevent the development of secondary cysts. Considering the rather frequent adverse effects of ABZ taken at the high dosage necessary to treat CE, and especially its liver toxicity often responsible for drug withdrawal in patients who depend on the drug for their survival (Horton, 2003), WZB117 may likely become an alternative drug to ABZ not only for CE but also AE for which ABZ use is inescapable.

Most of the candidate drugs that were promising, from *in vitro* and experimental studies, against CE and AE have not reached the pre-clinical stage because of adverse effects even superior to those of ABZ (Schein, 2020). Even though it is a promising strategy for the treatment of CE, we cannot ignore the potential adverse effects of WZB117 on the host. It has been previously reported that WZB117 inhibits facilitated glucose transport by competing with sugars for occupancy of the exofacial substrate binding site of the transporter (Ojelabi et al., 2016). Prolonged inhibition of glucose transport could compromise the normal cellular glycolysis of the host, affect host insulin secretion and cause host’s cerebral energy failure (Brockmann, 2009). Completing a thorough analysis of all possible adverse events was beyond the scope of this study; however, we conducted a preliminary safety assessment of WZB117 in its use to treat CE, in the murine experimental model. We found no abnormalities in body weight and blood glucose in our mice treated with WZB117 for 28 days. There were no significant changes in the biological parameters under study, especially regarding liver toxicity. Our treatment cycle was only 4 weeks; long-term safety still needs to be further evaluated, since hyperglycemia and lipodystrophy were reported after long-term administration of WZB117 (Liu et al., 2012); however, our data are very promising to launch pre-clinical studies on the peri-operative anti-parasitic treatment of CE, because 1 month is the usual recommended duration of treatment in this situation. The results of our bioinformatics analysis show that the EgGLUT1-ss gene sequence of *E. granulosus s.s.* differs significantly from those of unrelated species but has a close homology with EmGLUT1, derived from *E. multilocularis*; even closer sequences and similar biological functions may be expected for EgGLUT1 of all species within the *E. granulosus s.l.* cluster, but this should be specifically tested whenever the sequences of GLUT1 from other species become available, to ascertain the usefulness of the therapeutic approach targeting GLUT1 in CE whatever the species involved. The design of GLUT1 inhibitors more selective for the protein structure of EgGLUT1 should also be the focus of future developments on CE drugs to avoid interference with the host’s GLUT1 while conserving therapeutic efficacy.

Taken together, our study shows that EgGLUT1 is involved in glucose uptake of *E. granulosus s.l*, which provides insights to better understand the growth and development of *E. granulosus s.l* and its glucose metabolism function, and highly suggests that EgGLUT1 is a novel drug target for the treatment of CE.

## Data Availability Statement

All datasets generated and analyzed for this study are included in the article/**Supplementary Material**.

## Ethics Statement

All procedures carried out with animals were approved by the Ethical Committee of the First Affiliated Hospital of Xinjiang Medical University (IACUC-20130425012). At the end of treatment, all mice were euthanized to avoid the pain of animals to the greatest extent.

## Author Contributions

The experiments were performed by KA, MY, and JL. The manuscript was written by KA and MY. The data analysis and technical support were provided by NY, HL, and XB. The manuscript was reviewed and edited by DV, RL, and GL. The work was directed by RL and GL. All authors contributed to the article and approved the submitted version.

## Funding

This research was funded by the National Natural Science Foundation of China (82060373, 81760369, 81360251, 81860361 and 32060223); State Key Laboratory of Pathogenesis, Prevention and Treatment of Central Asian High Incidence Diseases Fund (SKL-HIDCA-2020-BC3); Opening Fund of Xinjiang key labratory (2020D04028); “Tianshan Cedar” Science and Technology Innovation Talents Support Plan of Xinjiang Uygur Autonomous Region (No. 2019XS13).

## Conflict of Interest

The authors declare that the research was conducted in the absence of any commercial or financial relationships that could be construed as a potential conflict of interest.

## Publisher’s Note

All claims expressed in this article are solely those of the authors and do not necessarily represent those of their affiliated organizations, or those of the publisher, the editors and the reviewers. Any product that may be evaluated in this article, or claim that may be made by its manufacturer, is not guaranteed or endorsed by the publisher.
