# Supplementary material for: EgGLUT1 Is Crucial for the Viability of Echinococcus granulosus sensu stricto Metacestode: A New Therapeutic Target?
Source: Front Cell Infect Microbiol. 2021 Nov 11;11:747739. doi: 10.3389/fcimb.2021.747739 (PMC8632494; doi:10.3389/fcimb.2021.747739)
Supplement: Supplementary file 1 [file DataSheet_1.docx]

**SUPPLEMENTARY MATERIAL**


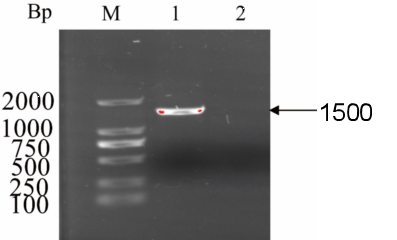


**Supplementary Figure 1.** Electrophoresis of the amplified PCR products of EgGLUT1-ss gene. EgGLUT1-ss gene fragment of 1500 bp was cloned from PSCs. M, DL2000; 1, EgGLUT1-ss gene; 2, Negative control.





**Supplementary Figure 2.** Cytotoxicity evaluation of WZB117 on primary mouse hepatocyte. The cell viability of primary mouse hepatocyte treated with WZB117 at concentrations (3.125, 6.25, 12.5, 25, 50 and 100 μmol/L) for 48 h, was detected by the CCK-8 assay. Values for each group were expressed as relative percentage of the DMSO group (1% DMSO) (one-way ANOVA).


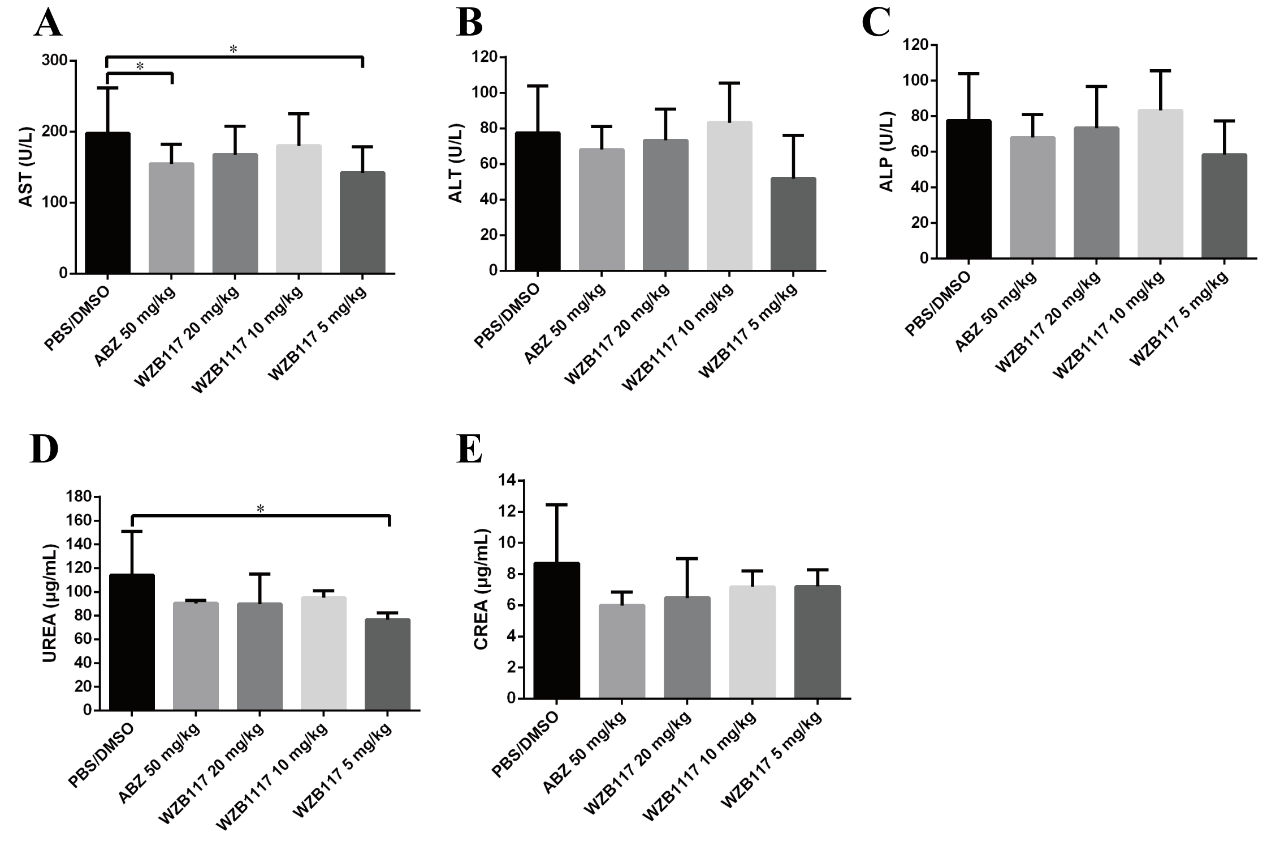


**Supplementary Figure 3.** Effects of WZB117 on biochemical indexes of *E. granulosus s.s.-*infected mice after 28 days of treatment. **(A)** The effects of WZB117 on AST content in mice. **(B)** The effects of WZB117 on ALT content in mice. **(C)** The effects of WZB117 on ALP content in mice. **(D)** The effects of WZB117 on UREA concentrations of mice. **(E)** The effects of WZB117 on CREA content in mice. PBS/DMSO (1:1, v/v) represent the control group. * Compared with the control group, the difference was statistically significant (* *P* < 0.05) (one-way ANOVA).

**
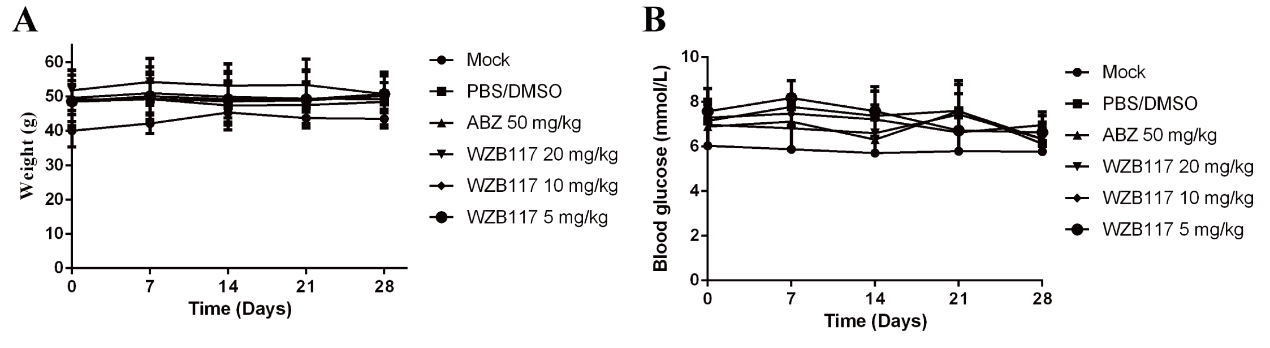
**

**Supplementary Figure 4.** Changes in body weight and blood glucose of *E. granulosus s.s.-*infected mice treated with WZB117. Mocks represent healthy mice. PBS/DMSO (1:1, v/v) represent the control group (one-way ANOVA).
